# DNA Methylation Biomarkers: Cancer and Beyond

**DOI:** 10.3390/genes5030821

**Published:** 2014-09-16

**Authors:** Thomas Mikeska, Jeffrey M. Craig

**Affiliations:** 1 Genetic Technologies Ltd., Fitzroy, Victoria 3065, Australia; E-Mail: thomas.mikeska@gtglabs.com; 2 Murdoch Childrens Research Institute, Royal Children’s Hospital, Parkville, Victoria 3052, Australia; 3 Department of Paediatrics, The University of Melbourne, Parkville, Victoria 3052, Australia

**Keywords:** cancer, diabetes, obesity, smoking, stress, autism, schizophrenia, bipolar disorder, depression, environmental factors

## Abstract

Biomarkers are naturally-occurring characteristics by which a particular pathological process or disease can be identified or monitored. They can reflect past environmental exposures, predict disease onset or course, or determine a patient’s response to therapy. Epigenetic changes are such characteristics, with most epigenetic biomarkers discovered to date based on the epigenetic mark of DNA methylation. Many tissue types are suitable for the discovery of DNA methylation biomarkers including cell-based samples such as blood and tumor material and cell-free DNA samples such as plasma. DNA methylation biomarkers with diagnostic, prognostic and predictive power are already in clinical trials or in a clinical setting for cancer. Outside cancer, strong evidence that complex disease originates in early life is opening up exciting new avenues for the detection of DNA methylation biomarkers for adverse early life environment and for estimation of future disease risk. However, there are a number of limitations to overcome before such biomarkers reach the clinic. Nevertheless, DNA methylation biomarkers have great potential to contribute to personalized medicine throughout life. We review the current state of play for DNA methylation biomarkers, discuss the barriers that must be crossed on the way to implementation in a clinical setting, and predict their future use for human disease.

## 1. Introduction

A biomarker is any biological characteristic that can be objectively measured and evaluated as an indicator of normal biological process, pathogenic process, or pharmacological response to a therapeutic intervention [1]. Biomarkers can be used at any stage of a disease and can be associated with its cause or latency (risk biomarkers), onset (diagnostic biomarkers), clinical course (prognostic biomarkers), or response to treatment (predictive biomarkers) ([2,3,4] and references therein). Biomarkers can also be associated with specific environments (exposure biomarkers). As almost all complex human diseases are caused by a mixture of genetic and environmental variation, biomarkers, especially those antecedent to disease, can be influenced by either of these factors. Biomarkers can also reflect the mechanisms by which exposure and disease are related. They can stratify individuals according to risk or prognosis and they can be used as targets or surrogate endpoints in clinical trials. An ideal biomarker must be able to provide clinically-relevant information, be accurately measurable in multiple individuals, ideally across multiple populations [2,4]. In this review we focus on DNA methylation biomarkers, review the current state of the field, and discuss limitations and our expectations for the future.

## 2. Epigenetics and Disease Latency

Epigenetics refers to the molecular marks that influence gene function in a mitotically-heritable manner [5]. Epigenetic marks are themselves influenced by a mix of genetic and environmental variation [6]. A typical gene will be regulated by epigenetic marks present at one or more gene promoters, which are usually but not exclusively close to its transcriptional start site, and by one or more enhancers, which can be within the gene or a large distance away from the gene [7]. Such regions of transcriptional control exhibit molecular characteristics in the form of multiple, synergistic epigenetic marks. 

Epigenetic marks include methylation of DNA at the cytosine residue of cytosine-phosphate-guanine (CpG) dinucleotides and covalent modifications of amino acid residues within histone proteins that are responsible for the primary packaging of DNA. Other cellular components, such as those involved in writing, reading, and erasing epigenetic marks, determine the local chromatin structure, which at two extremes can be open and active or closed and inactive [8].

In the human genome, DNA methylation occurs almost exclusively at CpG dinucleotides. The cytosine residue of a CpG dinucleotide can be covalently modified by adding a methyl group to its carbon-5 atom resulting in 5-methylcytosine. The methyl group is transferred from *S*-adenosyl-l-methionine to a cytosine residue via DNA methyltransferases (reviewed in [9,10]). CpG dinucleotides are unevenly distributed throughout the genome and are generally methylated [11]. Some CpG dinucleotides are clustered in regions known as CpG-islands, which can span hundreds to thousands of base pairs and are generally unmethylated [11].

The definition of a CpG island has been quite arbitrary and two algorithms have found widespread use throughout the scientific community to identify CpG-islands in genomic DNA sequences [12,13]. However, genome-wide studies have vastly increased our understanding of the human genome over the last few years, and more sophisticated algorithms for the identification of CpG-islands have been developed [14,15,16].

CpG islands are often, but not exclusively, located at gene promoters, where the methylation status is generally correlated with transcriptional gene activity [11]. DNA methylation can have other (regulatory) functions outside promoter regions, for example in intragenic regions [17,18], intergenic regions [19] and in regions of low CpG density [20]. DNA methylation performs a regulatory role at local and global levels. Global methylation is mainly determined by methylated CpG dinucleotides in highly repeated DNA sequences, such as satellite DNAs, which play an important function in maintaining genome stability [21]. DNA methylation level changes, namely local hypermethylation (gain of DNA methylation) and global hypomethylation (loss of DNA methylation), are often associated with a diseased state.

Most studies of the role of epigenetics in human disease have focused on investigating disease-associated DNA methylation changes and on determining the environmental influence on DNA methylation variations. Most of these have focused on cancer. It is now widely accepted that cancer results from a combination of genetic and epigenetic disruption or dysfunction (reviewed in [22]). Whereas the underlying causes of cancer remains largely elusive, it has also become clear that certain environmental factors such as the exposure to certain chemicals, toxins or heavy metals are capable of altering the epigenome and ultimately increase the risk of developing cancer [23,24,25].

Outside cancer, environmental influences on DNA methylation are the centre of the developmental origins of health and disease (DOHaD) phenomenon [26,27]. In this phenomenon, which grew out of the “fetal origins” hypothesis [28], adverse environment, *in utero* or in early postnatal life, programs the body for complex, non-communicable diseases including diabetes, cardiovascular disease (CVD) and neurodevelopmental disorders. Central to this phenomenon is the hypothesis that disease predisposition results when postnatal environment is mismatched to prenatal environment [29].

The DOHaD phenomenon involves a period of disease latency between the early origins and the later clinical manifestation. This latency may be in the form of a few years, for example with obesity and autism, or many decades, in the case of CVD. Non-epigenetic biomarkers of latent conditions such as CVD are already being developed and these include plasma high sensitivity C-reactive peptide, blood pressure, body mass index and artery wall thickness [30,31]. We discuss below how epigenetic biomarkers, in particular DNA methylation biomarkers, are being identified within the context of cancer and DOHaD.

## 3. Tissues and Bodily Fluids Suitable for Analysis of DNA Methylation Biomarkers

**Figure 1:**
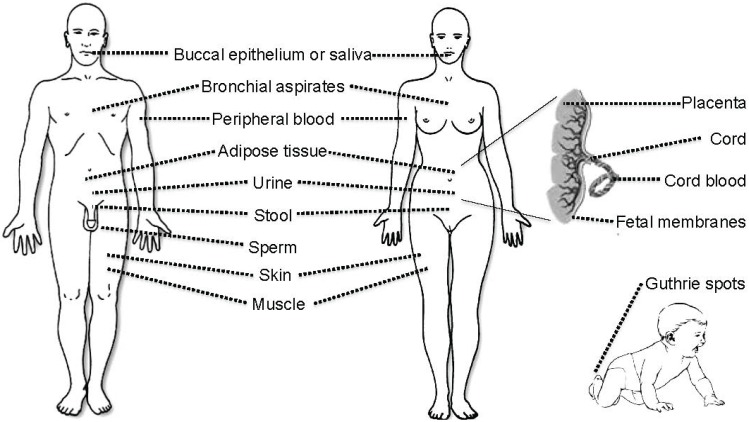
Illustration of the variety of tissues that can be used to investigate DNA methylation biomarkers. Note that tumor tissue is not listed individually as a cancer can affect any part of the body.

Almost any biological tissue sample or bodily fluid can be used for DNA methylation analysis. DNA methylation is the most robust epigenetic mark and will survive most sample storage conditions including, in the case of Guthrie neonatal blood spots, long-term drying [32]. DNA methylation can also be studied in histological specimens such as formalin-fixed paraffin-embedded (FFPE) samples [33] and microscopic preparations [34]. The robustness of DNA methylation marks makes DNA methylation analysis very attractive in a clinical environment as the analysis of gene expression pattern and histone modifications require more careful storage conditions, either with an RNA-preserving agent, by snap-freezing, or by cryopreservation of viable cells. In most cancers, (primary) tumor biopsies can be sampled but for the early detection of cancer and most other non-communicable diseases, only peripheral, easy-to-access tissues or bodily fluids can be collected. Such samples include venous peripheral blood, buccal epithelium or saliva, urine, stools, bronchial aspirates, and, even in some cases, muscle or adipose tissue [35,36,37,38,39] (Figure 1). At birth, placenta, umbilical cords and fetal membranes are also suitable tissues for analysis of DNA methylation [40,41,42].

It is important to note that even though it is desirable to measure disease-associated methylation biomarkers in a disease-relevant tissue, this condition does not always need to be met if a methylation biomarker is tightly associated with disease state. This is especially the case for tissues such as the brain and heart that can only be sampled *post mortem*.

Cellular homogeneity within a tissue is also a desirable characteristic for a DNA methylation biomarker [43]. Tissues such as blood or even blood fractions such as mononuclear cells, exhibit cellular heterogeneity [44,45,46]. However, methods have been developed to control for such heterogeneity, using either differential cell counts [47] or *post hoc* in regression models [48,49,50].

## 4. Parameters for Developing DNA Methylation Biomarkers

Before we go into more detail about specific DNA methylation biomarkers, we will review the measures of particular importance for assay performance and the barriers that must be breached in developing DNA methylation biomarkers. The nomenclature we use in this review is generally already in use, although it has not been previously summarized in such a way. It is as follows: single studies provide *potential biomarkers*, which could be *validated* using an independent technique and *replicated* in an independent cohort, also known as external validation. Following the systematic review and/or meta-analysis of a large number of independent studies, they become *candidate clinical biomarkers* that can enter clinical trials. Once approved, they become *proven clinical biomarkers* (Table 1).

**Table 1:** Nomenclature used in this review for the stages of DNA methylation biomarker development.

Nomenclature	Description
Potential biomarker	Results of a single study
Validated biomarker	Same finding using an independent method
Replicated biomarker	Same finding in independent cohort(s)
Candidate clinical biomarker	Replicated in multiple cohorts and subjected to systematic review and meta-analysis; most likely undergoing clinical trials
Proven clinical biomarker	Used in clinical practice

### 4.1. Methods for DNA Methylation Biomarker Discovery

Genome-wide profiling of DNA methylation patterns of healthy and diseased individuals has enabled the identification of potential methylation biomarkers for many diseases, most prominently in cancer but also other diseases such as metabolic or neurodevelopmental disorders. Following initial studies using pre-selected candidate gene approaches [51,52,53], many different genome-wide methods have been developed and used in the scientific community for DNA methylation biomarker discovery and good overviews are provided elsewhere [54,55,56,57,58]. Other scientific publications review such methods in the context of methylome-wide association studies (MWAS), which utilize a variety of platforms [59,60]. Typically, MWAS, as a subset of epigenome-wide association studies (EWAS), involves regression of DNA methylation at thousands to millions of CpG dinucleotides or CpG-rich regions on disease phenotype, outcomes or interventions. Such analyses usually adjust for multiple testing to produce potential methylation biomarkers in the form of differentially-methylated probes (DMPs) or regions (DMRs). Often, DMPs or DMRs are validated using locus-specific methods. The next stages of discovery following replication involve longitudinal analysis to resolve the issue of cause *vs.* effect in MWAS, and importantly to show whether replicated biomarkers can be used to predict a disease before its clinical onset or predict clinical outcomes after onset or after therapeutic intervention. Following discovery of such replicated biomarkers, further replication followed by meta-analysis and/or systematic review are required, at which stage these candidate clinical methylation biomarkers are ready for clinical trials leading to clinical proven methylation biomarkers. In this review we will focus on single locus DNA methylation biomarkers at all stages of discovery.

### 4.2. DNA Methylation Assay Sensitivity and Specificity

Assay sensitivity describes the proportion of patients with disease who have a positive test result (true positive rate), whereas the assay specificity describes the proportion of patients without disease who have a negative test result (true negative result) [61]. The ideal assay would show 100% sensitivity and 100% specificity. In other words, the test is never positive for a disease-free patient and never negative for a patient with disease. However, this ideal scenario is rarely achieved. It is also important to note that an assay with a sensitivity of 50% and a specificity of 50% is no better than tossing a coin to decide if the patient is harboring the disease or is disease-free [61].

The receiver operating characteristic (ROC) curve is a fundamental tool for diagnostic test or biomarker evaluation and visually displays the interdependency of specificity and sensitivity [62,63]. In a ROC curve the true positive rate (sensitivity; y-axis) is plotted in function of the false positive rate (1-specificity; x-axis). The area under the curve is equal to the probability that a classifier will rank a randomly chosen positive instance higher than a randomly chosen negative one. In other words, for a well performing diagnostic test or biomarker the curve is located towards the upper left corner. On the other hand a less well-performing diagnostic test or biomarker is characterized by a curve close to a diagonal line, representing a state in which sensitivity and specificity are similar.

It is desirable to achieve values for sensitivity and specificity as high as possible. However, for some tests it might be acceptable to achieve a higher sensitivity by sacrificing assay specificity or *vice versa*. This could be the case in particular for diseases for which a misclassification would result in severe consequences for the patient [61]. Acceptable values for sensitivity and specificity of a testing procedure can be determined by comparing to existing values of a test currently considered as gold standard. It is also important to consider that a diagnostic test is providing information independent of the experience of a clinician, which sometimes varies dramatically among hospitals and countries. However, it remains to be determined how easily the new testing procedure can be implemented in a clinical environment.

### 4.3. Barriers to Developing, Testing and Using DNA Methylation Biomarkers

Despite the promise of epigenetic biomarkers, so far only a few DNA methylation-based candidate biomarkers have reached the potential for use in a clinical setting, and all these are mainly related to the field of cancer. As with disease phenotypes, each clinical DNA methylation biomarker would need to be measured accurately and reproducibly. Differences in DNA methylation between cases and controls may be large (e.g., more than 50%) in cancer but in other non-communicable diseases may often be less than 5%. Methods used to measure methylation must be accurate to well below this level of resolution. The analytical sensitivity of specific methods is discussed below. Next, variability within the population needs to be small to maximize assay sensitivity and specificity. Predictive power also needs to be high. Positive predictive power is the percentage of people with a positive test who actually get the disease. These hurdles are all similar to those for any clinical trial.

## 5. Methods Suitable for the Analysis of Locus-Specific DNA Methylation Biomarkers

Many different methods have been described for the investigation of locus-specific DNA methylation (reviewed in [58,64,65,66,67]). Whereas some methods use genomic DNA for methylation analysis, the majority of methods require bisulfite-treated DNA as starting material [68,69]. Bisulfite treatment converts unmethylated cytosines to uracil, whereas 5-methylcytosines are relatively inert under reaction conditions. Subsequent use of bisulfite-treated DNA in PCR replaces the uracils with thymines and 5-methylcytosines with cytosines. Therefore, the methylation status of a particular CpG dinucleotide is detected indirectly [70].

The use of bisulfite-treated DNA has three important consequences for downstream applications for DNA methylation detection. Firstly, a considerable loss of initial input DNA can occur, due to extensive DNA degradation during the preparation and purification of bisulfite-treated DNA [71,72,73,74]. Loss of amplifiable DNA can be critical in particular for those samples where only a limited amount of genomic DNA is available, such as those from very small biopsies. Secondly, a poor bisulfite conversion rate can lead to false-positive results. This is of particular importance for very sensitive DNA methylation detection methods, such as those based on methylation-specific PCR (MSP) [75]. However, the use of a commercially-available bisulfite conversion kit can help to improve DNA recovery and to control for a proper bisulfite conversion rate [67,72]. Thirdly, PCR amplification may sometimes be biased towards unmethylated or methylated templates due to differences in CG content [76]. However, different approaches have been described in the literature to overcome or at least to minimize a potential PCR amplification bias [77,78,79,80].

Another problem for most downstream applications is the presence of heterogeneous DNA methylation patterns at many gene loci [81,82,83]. Heterogeneous methylation patterns are characterized by the presence of multiple epialleles (alleles which differ in the pattern of methylated and unmethylated CpG dinucleotides across the analyzed region). As every sample has its own set of epialleles, it can complicate quantification of methylation (reviewed in [84]) and cut-off value settings for when to call a sample unmethylated or methylated. The need for cut-off values also demands the use of quantitative DNA methylation detection methods, in particular for those gene loci, which are hypomethylated (loss of DNA methylation), or where already variable background methylation is present in healthy individuals [81,85].

Despite the many methodologies available for DNA methylation analysis the methodological considerations and requirements of a molecular diagnostics laboratory renders only a fraction of these methods suitable for DNA methylation analysis in a clinical setting. Such methods would need to use small quantities of DNA of varying quality. The latter is of particular importance for formalin-fixed paraffin-embedded (FFPE) specimens where the DNA is often degraded and chemically modified [86]. Ideally, DNA methylation detection methods for clinical settings should be low cost, easy to use, automatable, and capable of processing many samples in parallel in order to minimize costs of future tests. In the following sections we will discuss methods for DNA methylation detection suitable for use in clinical settings or in a molecular diagnostic laboratory.

Bisulfite pyrosequencing (Qiagen, Hilden, Germany) is based on sequencing-by-synthesis methodology and uses bisulfite-treated DNA as starting material [87,88,89]. This method is relatively cost- and time-effective, and is suitable for DNA methylation analysis of single gene loci. DNA methylation can be determined at single CpG dinucleotide resolution but methylation levels are provided in a quantitative manner for each CpG site as an average across all epialleles amplified during PCR. The analytical sensitivity is about 5%–10% for individual CpG dinucleotides [90,91]. This approach has a high-throughput capacity and is well suited for the analysis of small PCR amplicons, such as those typically generated from FFPE specimens. Importantly, this approach allows to quality control for a sufficient bisulfite conversion rate. However, the downside of this approach is that the instrument required to perform DNA methylation analysis is rather costly.

The MassARRAY EpiTYPER (Sequenom Inc., San Diego, CA, USA) also requires bisulfite-treated DNA as starting material and uses matrix-assisted laser desorption ionization time-of-flight (MALDI-TOF) mass spectrometry to extract (semi-) quantitative DNA methylation information from shifts and intensities of fragment signals after base-specific cleavage of PCR amplified epialleles present at single gene loci [92]. DNA methylation levels are determined as an average for a single CpG dinucleotide, or for multiple CpG dinucleotides, clusters of CpGs on the same fragment or for multiple CpGs across all fragments of amplicons generated during PCR [93]. Nevertheless, this approach is suitable for providing an almost complete methylation profile across the region-of-interest [92]. The analytical sensitivity is similar to bisulfite pyrosequencing [93] and DNA methylation data obtained by both methods for the same set of CpG dinucleotides has been shown to be highly concordant [81]. Like bisulfite pyrosequencing, MassARRAY EpiTYPER is suitable for high sample throughput and also requires the purchase of an expensive instrument.

Methylation-sensitive high-resolution melting (MS-HRM) is an inexpensive, fast and medium to high throughput screening methodology for DNA methylation analysis at single gene loci [94,95]. This approach requires bisulfite-treated DNA as starting material and exploits the differential melting behavior of PCR products generated from unmethylated and methylated epialleles. The melting profile of an unknown sample is compared to melting profiles of a DNA methylation standard series. This allows the reliably detection of homogeneous methylation levels down to 1%–5%, and can detect the presence of heterogeneous methylation patterns. However, the presence of heterogeneous DNA methylation allows the estimation of methylation levels in a semi-quantitative or qualitative manner. This is because the presence of heterogeneous DNA methylation results in a complex melting profile that does not allow the ready estimation of the amount of methylated epialleles; the result is largely qualitative [84]. MS-HRM is quite attractive in a clinical environment as PCR amplification and subsequent DNA methylation analysis is performed in one tube, which minimizes the risk of sample mix-up and sample cross contamination [96]. However, MS-HRM is not suitable on its own for use in a clinical setting as this method is not capable to deliver quantitative methylation information. Nevertheless, MS-HRM PCR products can be further quantified for DNA methylation using bisulfite pyrosequencing [97].

Another group of important approaches for DNA methylation detection is based on methylation-specific PCR (MSP) [98]. The strength of MSP-based approaches comes from the high analytical sensitivity, which allows them to detect only few methylated epialleles in a large background of unmethylated epialleles. The high analytical sensitivity originates from PCR primers containing CpG dinucleotides that selectively amplify only methylated epialleles. However, conventional MSP is not suitable for use in clinical settings as this approach detects DNA methylation only in a qualitative manner [98]. This can result in an overestimation of methylation in particular for those samples where background methylation is already present in normal tissues [81]. Moreover, conventional MSP is difficult to standardize between different laboratories and is also well known to generate false-positive, as well false-negative results, especially when DNA of low quality is used as starting material, for example FFPE-derived DNA [99,100]. Nevertheless, quantitative offshoots of conventional MSP, such as MethyLight [101], ConLight-MSP [102], MS-FLAG [103], SMART-MSP [104] and HeavyMethyl [105] are potentially suitable approaches for use in a clinical environment. The latter approach has been already successfully applied for DNA methylation detection of *SHOX2* and *PITX2* (see below).

Methylation-sensitive multiplex ligation-dependent probe amplification (MS-MLPA) plays a key role in the diagnosis of genomic imprinting disorders (see below) [106]. Different to the methods described above, this method uses genomic DNA as starting material to produce semi-quantitative DNA methylation information for single CpG dinucleotides. MS-MLPA relies on CpG dinucleotide-specific probes and a digestion step using the methylation-sensitive restriction endonuclease *HhaI* prior to PCR amplification to distinguish unmethylated from methylated epialleles. DNA methylation levels are determined by comparing peak sizes of patient samples with control samples and the analytical sensitivity is approximately 5%–20% [107,108,109,110]. MS-MLPA is suitable for high-throughput screening, is relatively cost-effective and does not require non-standard laboratory instruments as the PCR amplification products are separated by capillary electrophoresis on a DNA analyzer instrument.

The use of genomic DNA for methylation analysis is quite attractive as it avoids problems associated with bisulfite treatment. However, as MS-MLPA is based on a digestion step with a methylation-sensitive restriction endonuclease, false-positive results can occur as a result of incomplete digestion, in particular with DNA of poor quality. The use of the restriction endonuclease *HhaI* also limits the investigation of DNA methylation to *HhaI* recognition sites and therefore provides only a limited view of the DNA methylation landscape of any region of interest. However, MS-MLPA is capable of analyzing up to 50 CpG dinucleotides at any one time and allows the determination of DNA methylation levels at different gene loci simultaneously. Moreover, MS-MLPA can be combined with gene copy number and point mutation detection, which makes it a quite flexible methodology [110].

### Lessons Learned from the DNA Methylation Biomarker MGMT 

The DNA repair gene *O*^6^-methylguanine-DNA methyltransferase (*MGMT*) was first characterized in the early 1990s [111,112] and its key role in the resistance of malignant glioma to alkylating drugs was proposed repeatedly [113,114,115]. Approximately ten years later, a first link was established between *MGMT* methylation and improved patient outcome in response of malignant gliomas to the alkylating drug carmustine [116]. However, the relatively small number of patients investigated as well as some flaws in study design raised concerns of the validity of the results and warranted confirmation of the potential predictive biomarker *MGMT* (see comments in [116,117]).

Subsequently, *MGMT* methylation as a predictive biomarker of a patient’s response to alkylating drug regimens was replicated on different sample cohorts with mixed success. Methylation of *MGMT* was shown to serves as a predictive biomarker for determining response of glioma and glioblastoma patients treated with the alkylating agent Temozolomide [117,118]. Nevertheless, another study was not able to replicate *MGMT* methylation as a predictive biomarker in glioblastoma patients treated with alkylating drug regimens [119].

However, the seminal findings of a clinical trial reported in 2005, conducted by Hegi and colleagues, clearly showed that glioblastoma patients treated with Temozolomide showed a survival benefit if the promoter-associated CpG-island of the *MGMT* gene was methylated [120]. Since then, several clinical trials have confirmed *MGMT* methylation as a candidate clinical biomarker for determining patient response to Temozolomide treatment and it is now a proven clinical biomarker (reviewed in [121]).

Since 2005, many research groups and commercial companies (Table 2) have spent much effort developing assays to investigate the methylation status of *MGMT* by using various methods and platforms [122,123,124,125,126]. However, these methods varied in analytical sensitivity and provided methylation information ranging from purely qualitative to quantitative. As consequence, the general lack of consensus for an agreed methodology and the widespread use of inappropriate methodologies slowed down the implementation of *MGMT* methylation analysis in molecular diagnostics [127].

**Table 2:** Commercially-available DNA methylation test kits for cancer. References are either systematic reviews/meta-analyses ^1^ or a set of corroborating references ^2^. This table is an updated version of that shown in [127].

Gene(s)	Type of Biomarker	Type of Cancer	Diagnostic Test Kit: Brand Name (Manufacturer)	References
*VIM*	diagnostic	Colorectal	Cologuard (Exact Sciences)	[128] ^1^
*SEPT9*	diagnostic	Colorectal	Epi proColon (Epigenomics)	[129] ^1^
			ColoVantage (Quest Diagnostics)	
			RealTime mS9 (Abbott)	
*SHOX2*	diagnostic	Lung	Epi prolong (Epigenomics)	[130,131,132,133,134,135] ^2^
*GSTP1*/*APC*/*RASSF1A*	diagnostic	Prostate	ConfirmMDx (MDx Health)	[136,137,138] ^1^
*MGMT*	predictive	Glioblastoma	PredictMDx Glioblastoma (MDx Health)	[121,139,140] ^1^
			SALSA MS-MLPA probemix ME011 Mismatch Repair genes (MRC-Holland)	
			PyroMark MGMT Kit (Qiagen)	

Several recent studies assessing the clinical utility of different methodologies for *MGMT* methylation detection favor quantitative approaches such as bisulfite pyrosequencing [141,142]. Quantitative approaches are necessary to determine cut-off values for methylation ranges related to clinical information such as prognosis [143,144]. However, methylation cut-off values are not universal for a particular gene and strongly depend on the method used for DNA methylation analysis. Even by using the same methodology for methylation analysis requires determination of cut-off values for each assay as these values also depend on the region of the gene investigated, PCR primers and PCR conditions used as well as minimal tumor content required [143,144,145]. It has also been recognized that careful studies of the entire *MGMT* promoter-associated CpG-island are required to determine those CpG dinucleotides or CpG clusters suitable as a surrogate biomarker for biological or clinical relevant information [83,146].

Keeping in mind that *MGMT* methylation was one of the first DNA methylation biomarkers to be identified, it is not surprising that it took a considerable amount of time until it found its way into the clinic. Advancements in study and clinical trial design will certainly help to speed up replication and clinical implementation of new DNA methylation biomarker. However, the current lack of an agreed methodology as the gold standard for DNA methylation analysis is still a roadblock to overcome. For a more detailed view on which milestones need to be achieved in bringing a DNA methylation biomarker into clinical practice we refer the interested reader elsewhere [127].

## 6. DNA Methylation Biomarkers

To date, the vast majority of replicated and candidate clinical DNA methylation biomarkers come from cancer research. Clinically-relevant DNA methylation biomarkers outside cancer exist for diseases originating from genomic imprinting disorders, such as Prader-Willi and Angelman syndrome (see below), and are currently being developed for a wide range of environmental agents and the chronic diseases to which they predispose. The following sections will give an overview of promising DNA methylation biomarkers for potential clinical use.

### 6.1. Candidate Clinical DNA Methylation Biomarkers for Cancer

A selection of candidate clinical DNA methylation biomarkers for cancer will be discussed below; many others have been described in greater detail elsewhere (e.g., [127,147,148,149,150,151,152,153,154,155,156,157,158,159,160,161]) or have been subject of systematic reviews and meta-analysis (e.g., [129,136,137,138,139,140,162]). Not surprisingly, much effort has been spent in identifying diagnostic DNA methylation biomarkers suitable for early detection and diagnosis of cancer. Early detection allows treatment of the cancer at a stage that is generally considered beneficial for disease outcome. Such tests could be blood-based or use other bodily fluids collected less invasively, which makes it very convenient to the patient. Prognostic biomarkers would provide information on a patient’s overall survival if the disease is left untreated, whereas predictive biomarkers would be suitable for determining a patient’s response to a certain drug regimen. The latter are of particular importance as they may help to minimize the health burden of patients, as well as to minimize costs for healthcare providers for unnecessary drug treatment.

DNA methylation-based candidate clinical biomarker genes for the early detection include vimentin (*VIM*) [128,163], septin 9 (*SEPT9*) [129,164], and syndecan 2 (*SDC2*) [165,166] for colorectal cancer, glutathione S-transferase pi 1 (*GSTP1*) for prostate cancer [136,167,168], and cyclin-dependent kinase inhibitor 2A (*CDKN2A*) [169,170] and short stature homeobox 2 (*SHOX2*) (see below) for lung cancer. These have already reached clinical potential and for some diagnostic test kits are commercially-available (Table 2). In the next sections we will provide an overview of *SHOX2*, *PITX2* and *MGMT* as good examples of diagnostic, prognostic and predictive biomarkers in cancer.

#### 6.1.1. *SHOX2*

DNA methylation of the short stature homeobox 2 (*SHOX2*) gene was found to be a diagnostic clinical biomarker candidate for the detection of malignant lung disease even in patients where histology and cytology results are equivocal [135]. *SHOX2* methylation allowed the specific detection of malignant lung disease with a sensitivity of 60% and a specificity of 90% in blood plasma using HeavyMethyl, a quantitative methylation-specific PCR-based approach [134]. The highest assay sensitivity was achieved for small cell lung cancer (SCLC) cases with 80% and squamous cell carcinoma (SCC) with 63%, respectively, when compared to adenocarcinomas (AC) cases with a sensitivity of only 39%. However, the poor sensitivity for detecting adenocarcinomas could be improved by the addition of a second (or more) adenocarcinoma-specific biomarker. Not surprisingly, the sensitivity values obtained of the blood-plasma-based assay were lower compared to sensitivities seen from bronchial aspirates (SCLC: 97% (80%), SCC: 82% (63%), and AC: 47% (39%)); overall sensitivity and specificity were 68% (60%) and 95% (90%), respectively) as the tumor-derived amount of DNA is expected to be lower in blood than a lung-specific analyte [134,135]. However, a blood-based assay has the advantage of using specimens which have been collected with a far less invasive procedure compared to those obtained from bronchoscopy. Furthermore, a blood-based assay enables screening of asymptomatic patients whereas availability of bronchoscopy is limited to patients with suspected lung cancer. Noteworthy, elevated *SHOX2* methylation levels in pleural effusions do not only allow the detection of lung cancer but also the detection of other malignancies, such as breast cancer and gastrointestinal cancers [132,133]. However, assay sensitivity and specificity for these was not as good as for bronchial aspirates or blood. *SHOX2* methylation level in lymph node tissue obtained by endobrochial ultrasound with transbronchial needle aspiration (EBUS-TBNA) improved endoscopic lung cancer staging with an assay sensitivity and specificity of 94% and 99%, respectively [130].

DNA methylation of *SHOX2* not only provides diagnostic but also provides prognostic information for cancer patients [131,132]. Pleural effusion samples obtained from patients with different malignancies (including lung cancer) showed a shorter overall survival if elevated levels of *SHOX2* methylation were detected [132]. Contrarily, gain of *SHOX2* methylation in tumor tissues has been shown to be associated with good prognosis in lung cancer patients. The prognostic power of *SHOX2* methylation was further improved when combined with DNA methylation analysis of *PITX2* [131].

#### 6.1.2. *PITX2*

The paired-like homeodomain 2 (*PITX2*) gene encodes the PITX2 transcription factor. DNA methylation status of the *PITX2* promoter has been identified as a candidate clinical biomarker in tumor tissues. This has provided prognostic information for breast cancer, prostate cancer, and lung cancer. *PITX2* methylation in estrogen receptor alpha positive breast cancer patients without lymph node metastasis has been associated with poor prognosis when treated without any systemic adjuvant therapy [171] as well as a higher risk of disease recurrence after surgery when treated with the antiestrogen Tamoxifen only [172,173]. Furthermore, methylation of the *PITX2* promoter was also associated with poor patient outcome in estrogen receptor alpha positive, HER-2/*neu*-negative breast cancer patients positive for lymph node metastasis when treated with an anthracycline-based adjuvant chemotherapy [174]. Methylation of *PITX2* in prostate cancer patients has also been shown to be a prognostic biomarker for an increased risk of biochemical recurrence after radical prostatectomy [175,176,177]. Importantly, the prognostic value of *PITX2* methylation was particularly high in tumor-enriched samples of patients at intermediate risk for whom further risk stratification is quite often challenging [176]. Interestingly, and different to breast and prostate cancer, increased DNA methylation levels of *PITX2* were associated with prolonged survival in lung cancer patients and requires further investigation [131].

#### 6.1.3. *MGMT*

*O*^6^-methylguanine DNA methyltransferase is a DNA repair protein that is encoded by the *MGMT* gene and is capable of removing alkyl residues directly from the *O*^6^-position of guanines. However, if the DNA repair capacity of *MGMT* is impaired or inactivated, for example by DNA methylation, affected cells are less protected against mutagenic DNA adducts [178,179]. Therefore, tumor *MGMT* promoter methylation renders cancer cells susceptible to the cell damaging effects of drug regimens utilizing alkylating agents [116,180] (see also *Lessons learned from the DNA methylation biomarker MGMT*). *MGMT* was one of the first predictive DNA methylation biomarkers to determine a patient’s response to alkylating chemotherapeutics and it was shown that glioblastoma patients with tumor *MGMT* promoter methylation have a survival benefit from Temozolomide chemotherapy [120,121].

The more frequent use of quantitative approaches such as bisulfite pyrosequencing to detect and measure *MGMT* methylation have revealed that the DNA methylation biomarker *MGMT* does not only have a predictive but also a prognostic clinical component (reviewed in [121,126]). Glioblastoma patients with more than 29% *MGMT* promoter methylation showed a longer progression-free and overall survival when treated with radiotherapy and Temozolomide [143]. A methylation cut-off value of 25% separated elderly glioblastoma patients into two groups with those having more than 25% of methylation had a better survival rate when treated with alkylating agents alone [144]. Tumor *MGMT* methylation status was also shown to have a prognostic value for progression-free survival of anaplastic glioma patients treated with radiotherapy alone [181,182].

## 7. DNA Methylation Biomarkers for Genomic Imprinting Disorders

Whereas most genes are expressed from both the maternal and paternal allele, imprinted genes are monoallelically expressed in a parent-of-origin-specific manner either from the maternal or the paternal allele. Only a small proportion of all human genes are imprinted and are often found clustered in imprinted domains and mono-allelic gene expression is controlled by differentially methylated regions (DMRs) (reviewed in [183]). Disrupted or altered imprinting patterns have been linked to pathological conditions termed genomic imprinting disorders (reviewed in [184]). Examples of imprinting disorders include Prader-Willi syndrome (PWS), Angelman syndrome (AS), Beckwith-Wiedemann syndrome (BWS) and Silver-Russell syndrome (SRS), which will be discussed briefly below. 

PWS and AS are clinically distinct neurodevelopmental imprinting disorders, which have been linked to a region on the long arm of human chromosome 15 (15q11–q13; reviewed in [185]). This region consists of several imprinted genes and the absence of paternally expressed genes in this imprinting domain results in PWS, whereas the loss of maternally-expressed genes causes AS. Additionally, point mutations in the E6-AP ubiquitin-protein ligase (*UBE3A*) gene, which is also part of the imprinting domain account for approximately 10% of AS patients. In cases where PWS or AS is suspected, DNA methylation analysis of the PWS/AS critical region allows the reliable identification of more than 99% of PWS patients and about 80% of AS patients [186]. 

Two approaches are commonly used in molecular diagnostics for DNA methylation analysis of the PWS/AS critical region [186,187]. The first approach determines the methylation status at a single gene locus, the small nuclear ribonucleoprotein polypeptide N (*SNRPN*) gene, whereas the second approach determines the methylation status and gene copy number changes at several sites across the region [186]. DNA methylation analysis of the *SNRPN* gene is frequently determined by MSP [188,189] whereas the simultaneous investigation of methylation levels and gene copy numbers is determined by methylation-sensitive multiplex ligation-dependent probe amplification (MS-MLPA) [190]. Molecular diagnostics of PWS and AS is quite complex and challenging, and guidelines for molecular genetic testing and reporting PWS and AS have been developed [186]. Furthermore, a WHO international genetic reference panel for PWS and AS has been established and was successfully validated in an international multicentre study [187].

BWS and SRS are imprinting disorders, which have been associated with imprinted genes on chromosome region 11p15.5 [191,192,193]. This region is functionally divided into two domains: the first domain consists of the imprinted insulin-like growth factor gene 2 (*IGF2*) and the non-coding RNA *H19* and is controlled by DMR1 whereas the second region contains several imprinted genes, including cyclin-dependent kinase inhibitor 1C (*CDKN1C*), potassium voltage-gated channel, KQT-like subfamily, member 1 (*KCNQ1*) and KCNQ1 opposite strand/antisense transcript 1 (*KCNQ1OT1*), is controlled by DMR2. Loss of methylation at DMR2 (*KCNQ1OT1* hypomethylation), is the most frequent alteration, in around 50% of BWS patients [194] whereas loss of methylation at DMR1 (*H19* hypomethylation) is typically observed in SRS is found in around 40% of SRS patients [192,195]. As mentioned before, MS-MLPA allows the simultaneous investigation of methylation levels and gene copy numbers and has thus been considered well suited for detecting the majority of (epi-) genetic alterations associated with BWS and SRS in region 11p15.5 [196,197,198].

Most approaches for routine clinical DNA methylation analysis at single-gene loci in genomic imprinting disorders rely, most probably for historical reasons, on qualitative methylation detection methods. However, the diagnostic advantages of quantitative DNA methylation detection methodologies, such as bisulfite pyrosequencing [191,199,200], are being increasingly recognized and will be probably the preferred methods of choice for analyzing single gene loci in the near future.

## 8. DNA Methylation Biomarkers of Outcome in Chronic Diseases Other than Cancer

Given the likely early life origins for non-communicable disease, there are plenty of opportunities in which DNA methylation biomarkers could be used. Biomarkers for intrauterine environmental exposures such as maternal alcohol consumption or smoking could provide a way to measure exposures without the need for time-consuming, hard-to-administer questionnaires and where access to mothers is not possible. DNA methylation risk biomarkers could be used to stratify risk for latent non-communicable disease before the onset of disease. They could also be used to monitor progression from first symptoms to full disease. After disease onset, they could be used for predicting survival and response to therapy as they are beginning to do with cancer. Below, we review data from the most promising studies of environmental, risk, diagnostic, prognostic, and predictive DNA methylation biomarkers.

### 8.1. DNA Methylation Biomarkers for Adverse Environments

There have been a large number of environmental agents linked to epigenetic change, including toxins, stress and nutrition, and these have been reviewed elsewhere [201,202,203]. Below, we focus on two that have yielded replicated DNA methylation biomarkers: smoking and stress.

#### 8.1.1. *AHRR* Methylation and Smoking

Exposure to adverse environments at all stages of life have been shown to influence the epigenome (reviewed in [39,42,204,205]). However, a replicated association has been found for only one: the effect of DNA methylation on the aryl hydrocarbon receptor repressor (*AHRR*) gene involved in the detoxification of chemicals found in tobacco smoke. As of June 2014, ten independent methylome-wide studies using Illumina Infinium HM450 arrays (containing probes for about 480,000 CpG dinucleotides located in functionally-relevant regions of the genome [206]) had all identified the same smoking-associated probe, cg05575921, located in a region of intermediate CpG density (CpG-island shore) 450 bp upstream of a CpG island in the third intron of the *AHRR* gene [207,208,209,210,211,212,213,214,215,216] (Table 3). Two studies focused on the effect of maternal smoking in umbilical cord blood [209,215], which they and others [217] replicated in independent sample cohorts. Others found an association of adult smoking with *AHRR* methylation in blood [207,208,210,211,212,214], lung tissue [211] or blood lymphoblastoid cell lines [213]. No effects were seen at birth in placenta or buccal epithelium [217] and effects were seen elsewhere in the *AHRR* gene in lung alveolar macrophage DNA but not at the cg05575921 probe [213]. Three studies performed within-cohort validation using locus-specific DNA methylation analysis [207,211,212] and six studies replicated their findings in adults in independent cohorts [208,209,211,212,214,215]. Two studies showed evidence of a role for the region surrounding probe cg05575921 in regulation of *AHRR* expression [211,213]. All found an inverse relationship between smoking and DNA methylation with an effect size ranging from −4% in neonates of mothers who smoked throughout pregnancy [215] to −24.4% in adult current smokers [212]. 

Similar effects were seen in Europeans, African Americans [207], and South Asians [208]. The latter study found that current smokers were identified with 100% sensitivity and 97% specificity in Europeans and with 80% sensitivity and 95% specificity in South Asians. Timing-specific effects were also found; prenatal smoking only exerted an effect when mothers smoked during a significant part of gestation [217,218]. Furthermore, associations found at birth were also present at 18 months of age [217] but in adulthood, DNA methylation levels were similar in never smokers and in former smokers [212]. Clearly, loss of methylation at and around the *AHRR* cg05575921 probe is strongly associated with first or second hand exposure to smoking. Importantly, one study found an association in adults with smoking, but not tobacco snuff consumption, implicating that a product(s) of tobacco combustion is responsible for the loss of DNA methylation rather than tobacco itself. Further work is needed to link this loss to the timing of prenatal smoking, and postnatal passive and active smoking, and its relationship with downstream health outcomes previously associated with *AHRR* polymorphisms such as cancers [219,220,221] and endometriosis [222].

In addition to probe cg05575921, a number of CpG dinucleotides have been significantly associated with prior smoking. Table 4 lists these probes, using a cut-off of those that have been identified by four or more studies. These include two further CpG dinucleotides from *AHRR* [207,209,210,211,212,213], one from the thrombin receptor-like 3 (*F2RL3*) gene [208,211,214,216], one from the growth factor independent 1 transcription repressor (*GFI1*) gene and two from the myosin 1G (*MYO1G*) gene. In addition, two intergenic smoking-associated CpG sites have been replicated across multiple studies [207,208,209,211,212,213,214], all coinciding with regions of DNAse hypersensitivity, suggesting functional significance. Potentially, one or more of these CpG dinucleotides could be used in combination with probe cg05575921 as DNA methylation biomarkers for smoking. 

**Table 3:** Summary of findings for the relationship between smoking and DNA methylation within the *AHRR* gene. Data refer to *AHRR* HM450 probe cg05575921 unless otherwise stated. Summary includes details of assay platform, age of subjects, details of exposure, tissues examined, number of subjects, whether *AHRR* expression was also measured, whether findings were validated or replicated and effect size (methylation levels in smokers compared to non-smokers).

Reference	Platform	Age, Median	Exposure	Tissue	N	Effects Elsewhere in AHRR	*AHRR* Expression	Vali-dation	Repli-cation	Effect Size	Notes
[213]	HM450	Adults, 45	Current smoking	LCLs & alveolar MP	119/19 ^1^	yes	Yes ^2^	No	No	−15%/NS	
[209]	HM450	Birth	Maternal smoking ^3^	Whole CB	1062/36 ^4^	Yes	No	No	Yes ^4^	−7.5%/−7.7% ^4^	Multiple hits in the aryl hydrocarbon signaling pathway. Authors have since shown that effects are specific for maternal smoking through at least gestational week 18 [218]
[207]	HM450	Adults, 49	Current smoking	PBMC	111	Yes	No	Yes	No	−15%	African Americans
[208]	HM450	Adults, 48	Current smoking	Whole PB	81/84 ^5^	No	No	No	Yes ^6^	−22%	Former smokers same as never smokers; changed only slightly after adjusting for cell composition
[210]	HM450	Adults, 22	Current serum cotinine	PBMC	107	yes	No	No	No	−20% ^7^	
[211]	HM450	Adults, 51/55/49/? ^8^	Current smoking	Whole PB, lung tissue	184/190/180/27	yes	Yes ^9^	Yes	Yes	−17%/−14%/NS/NS ^10^	Replicated in a mouse model of smoking exposure
[212]	HM450	Adults, 60/53 ^11^	Current smoking	Whole PB	749/232 ^11^	yes	No	Yes	Yes ^11^	−24/−23% ^11^	methylation-specific protein binding patterns were observed for cg05575921; levels in former smokers revert to levels similar to never smokers over time
[215]	HM450	Birth	Maternal smoking	Whole CB	889	yes	No	No	Yes	−4%	Replicated a previous study [209]
[214]	HM450	Adults, 43	Current smoking	Whole PB	432	yes	No ^13^	No	Yes	−7.4%	Replicated a previous study [212]; no effect with tobacco snuff
[216]	HM450	Female adults, 57	Current smoking	Whole PB	200	No	No	No	Yes	−8%	Former and never smokers had similar methylation levels
[217]	Sequenom EpiTyper	Birth & 18 months	Maternal smoking	CBMC, buccal epithelium, placenta	46/15/24 ^12^	yes	Yes ^14^	n/a	Y	−10%/NS/NS ^12^	No effect if mother smoked early pregnancy only; effects of smoking stable to 18 months of age

^1^ refers to the two different cell types tested; ^2^ *AHRR* expression in alveolar macrophages was inversely correlated with methylation of probe cg05575921; ^3^ measured using plasma cotinine at gestational week 18; ^4^ replicated using data from maternal smoking in pregnancy in an independent cohort; ^5^ data on Europeans replicated in South East Asians; ^6^ replicated across two ethnic groups; ^7^ effect size calculated from the regression line, highest to lowest plasma cotinine; ^8^ discovery, replication and validation groups are subsets of the same cohort and were analyzed along with lung tissue samples from a separate cohort; ^9^ *AHRR* expression in lung tissue was inversely correlated with methylation of probe cg05575921; ^10^ no difference with probe cg05575921, differences found for *AHRR* probes cg21161138 and cg23576855 (magnitudes similar to those seen in blood); ^11^ discovery and replication subsets of the same cohort; ^12^ significant associations between methylation and expression seen at six genes other than *AHRR*; ^13^ CBMC/buccal epithelium/placenta; ^14^ *AHRR* expression non-significantly higher in CBMCs in newborns exposed to smoking in pregnancy than those not exposed. Abbreviations: LCL, lymphoblastoid cell lines; MP, macrophages; PB, peripheral blood; CB, cord blood; MC, mononuclear cells; NS, not significant.

**Table 4:** Other HM450 probes with significant correlations with smoking in at least four studies. Probes are included if found to be significantly associated with smoking in at least four independent studies. DHS, DNAse hypersensitive site, indicative of regulatory potential.

Probe	Gene	References
cg03991871	*AHRR*	[209,212,213,215]
cg21161138	*AHRR*	[207,209,210,211,212,215]
cg03636183	*F2RL3*	[208,211,214,216]
cg09935388	*GFI1*	[208,209,212,214,215]
cg22132788	*MYO1G*	[208,209,210,214]
cg12803068	*MYO1G*	[210,212,215,218]
cg21566642	Intergenic (CpG island, DHS)	[207,208,211,212]
cg06126421	Intergenic (enhancer, DHS)	[207,208,211,212,214]

#### 8.1.2. *NR3C1* Methylation and Stress

Stress triggers the activation of the hypothalamus-pituitary-adrenal axis, resulting in the production of glucocorticoids by the adrenal glands. By binding to receptors in the brain, glucocorticoids induce changes in gene expression and in turn, health and behavior [223]. Landmark studies with rats have shown that lack of maternal licking and grooming at birth resulted in an increased level of DNA methylation within the exon 1_7_ promoter of the glucocorticoid receptor gene *Nr3c1* in rat hippocampus, in particular at a region that binds nerve growth factor-inducible protein-A (NGFI-A) [224,225]. Since then, studies of the equivalent region in humans (exon 1F of the *NR3C1* gene) have found decreased DNA methylation in cord blood [226,227] and placenta [228] associated with maternal anxiety during pregnancy. Others have shown that violence towards women during pregnancy can have a similar effect [229,230]. Even extremes of stress experienced prior to conception, in the form of the holocaust, were also found to correlate with *NR3C1* exon 1F methylation, albeit in opposite directions depending on the sex of the parent [231]. Methylation analysis of various tissues from adults, either alive or *post mortem*, have found long-lasting effects of abuse [232,233,234,235] or death of a parent [235,236] during childhood on *NR3C1* exon 1F. In addition, adults with post-traumatic stress disorder had decreased DNA methylation at the same [237] or alternate [238] *NR3C1* promoters. Of further interest, three studies have shown that methylation of *NR3C1* exon 1F can predict health outcomes, whether predicting quality of movement and attention at birth [239], response to psychotherapy in adults with posttraumatic stress disorder [240] or response to threat-associated stress in adult females [241]. In the latter study, DNA methylation levels at *NR3C1*, the estrogen receptor alpha gene *ESR1* and the serotonin transporter gene *SL6A4* each had independent predictive power. Furthermore, a model containing data from all those genes accounted for half of the variance in total cortisol output. Rat studies showing that the adverse effects and DNA methylation changes associated with early neglect could be reversed in adulthood by methyl-donor rich diet [242] or the histone deacetylase inhibitor Trichostatin A [243], suggesting that *NR3C1* methylation could be use to monitor response to future interventions in humans.

Clearly, methylation at *NR3C1* promoters has the potential to be developed into a variety of candidate biomarkers. In addition, despite yielding no replicated stress biomarkers to date, the small (typically 1%–2%) effect sizes for *NR3C1* methylation would suggest that there may be better DNA methylation-based stress biomarkers out there, discoverable using epigenome-wide approaches [244,245,246,247,248].

### 8.2. DNA Methylation Risk Biomarkers at Birth

Measuring DNA methylation in five candidate genes in DNA from umbilical cords, Godfrey and colleagues found that methylation of two genes correlated with childhood adiposity as measured by fat mass and trunk/limb fat ratio in 78 nine-year-olds [42]. Methylation of the retinoic acid X receptor alpha (*RXRA*) gene and the endothelial nitric oxide synthase (*ENOS*) gene, together with sex, explained 25% of the variance in adiposity at age nine. Data for *RXRA* were replicated in a second cohort of 239 six-year-olds [42]. Other studies have identified associations between *RXRA* methylation in cord blood at birth and bone mineral density at age four [249] and between methylation of the alkaline phosphatase *ALPL* and body mass index at nine years of age [250]. However, the first association could not be replicated in another sample cohort whereas for the second association no replication study was performed.

### 8.3. DNA Methylation Biomarkers during Childhood

Rakyan and colleagues identified 132 CpG dinucleotides whose methylation levels differed significantly in twin pairs discordant for type 1 diabetes and which were subsequently validated with an independent method and replicated in a further set of twin pairs [251]. Two-thirds of these CpG dinucleotides were also present in singletons prior to the onset of overt symptoms of type 1 diabetes but positive for diabetes-associated autoantibodies. If those findings can be further replicated, this could provide single or panels of DNA methylation candidate clinical biomarkers predicting the onset of type 1 diabetes. A potential biomarker study found that DNA methylation within the promoter of the peroxisomal proliferator activated receptor gamma (*PPARG*) gene in blood at age five to seven years predicted obesity risk from nine to 14 years [252]. However, these results have yet to be replicated.

Autism spectrum disorder (ASD) describes a related set of neurodevelopmental disorders of childhood characterized by social deficits and communication difficulties, stereotyped or repetitive behaviors and interests, and in some cases, cognitive delays. To date, a small number of ASD MWAS have been performed, using a variety of platforms, on lymphoblastoid cell lines [253], peripheral blood [254,255], buccal epithelium [256], *post mortem* occipital cortex and cerebellum [257], and dorsolateral prefrontal cortex, temporal cortex and cerebellum [258]. ASD-specific DNA methylation was found in all but one study [257] and in the rest, although ASD-specific methylation was often validated within the study, only one study attempted to replicate across cohorts and tissues [258]. In this study, three significant ASD-associated array probes discovered in temporal cortex were replicated in such a manner. ASD-specific DNA methylation found within the proline-rich transmembrane protein 1 (*PRRT1*) gene was replicated in prefrontal *post mortem* cortex and cerebellum, methylation of *c11orf21* was replicated in prefrontal cortex and methylation at an intergenic site near the zinc finger gene *ZFP37* was replicated in a sex-specific manner in cerebellum. The only differentially methylated gene replicated in two separate studies is the olfactory receptor gene *OR2L13* found in buccal epithelium [256] and peripheral blood [254]. Further replication will be required to develop this potential biomarker for ASD.

### 8.4. DNA Methylation Biomarkers in Adults

Cardiovascular disease (CVD) and its precursors are receiving arguably the greatest attention in MWAS outside cancer [259,260,261]. DNA methylation biomarkers could help ascertain risk early in life, help with diagnosis and predict response to interventions. Below, we report some of the more advanced such studies. 

Levels of fasting glucose and insulin and measures of insulin resistance are used to test for early signs of diabetes and they have been subject to a recent MWAS [262]. This study divided up a cohort of 837 non-diabetic individuals at a median age of 48 years into discovery and replication subsets. Using HM450 arrays on DNA from CD4+ T cells, the investigators found significant associations between methylation of two CpG sites with the ATP-binding cassette gene *ABCG1*, involved in macrophage cholesterol and phospholipids transport, with insulin resistance, with one associated with insulin “of borderline significance”. The CpG site with the strongest association with insulin and insulin resistance was also strongly associated with nearby single-nucleotide polymorphisms, implying that differences in genetic sequence can alter the epigenetic functionality of a genomic region. Another recent study replicated across two cohorts a DNA methylation biomarker for triglyceride levels at the carnitine palmitoyltransferase gene *CPT1A* in the same cell type [263]. In this study, *CPT1A* methylation explained 11.6% and 5.5% of the variation in triglyceride levels in the discovery and replication cohorts, respectively.

Although several studies have discovered associations between DNA methylation and obesity [264], few studies have searched for risk or predictive DNA methylation biomarkers in adulthood. In one study that did, males with a history of CVD had higher global DNA methylation than those without [57]. Those who went on to develop symptoms of CVD six years later had intermediate levels of global DNA methylation. In other study, a type 2 diabetes-specific CpG dinucleotide in the first intron of the fat mass and obesity-associated gene *FTO* predicted the onset of symptoms between ages 30 and 43 in a cohort of initially asymptomatic adults [265]. Replication is required for both studies.

Two unreplicated studies resulted in potential predictive DNA methylation biomarkers for response to weight loss programs in adults. In the first, obese women with better response to dietary intervention showed significantly lower levels of DNA methylation at promoters of the leptin (*LEP*) and TNF-alpha (*TNF*) genes than the non-responder group [266]. Although no differences were found between responder and non-responder groups in *LEP* and *TNF* gene expression, if replicated, the potential predictive methylation biomarker would still be valid on its own. In a similar study of obese men, DNA methylation levels in several CpG dinucleotides located in the ATPase *ATP10A* and the CD44 antigen (*CD44*) genes showed statistical baseline differences depending on the weight-loss outcome [266]. Again, these finding have not yet been replicated.

In a search for potential DNA methylation biomarkers of postpartum depression using MWAS and a parallel study in mice, Guintivano and colleagues found that DNA methylation at the heterochromatin protein 1 binding protein 3 (*HP1BP3*), and tetratricopeptide repeat domain 9B (*TTC9B*) genes predicted postpartum depression in the original and replication cohorts [267]. Adjustment for blood cell heterogeneity resulted in a higher specificity (96%) in both cohorts compared to unadjusted values.

Schizophrenia is a psychotic disorder, and bipolar disorder is a mood disorder but both have similar symptoms and they are often studied together. Many potential DNA methylation biomarker studies and MWAS have been conducted for these disorders ([268,269,270,271,272,273,274] and references therein). Despite the heterogeneity of platforms and tissues used in these studies, a small number of potential diagnostic schizophrenia- and/or bipolar disorder-associated biomarkers have been identified. The serotonin receptor 2A (*HTR2A*) gene was differentially methylated in both disorders in two brain regions (frontal cortex and the anterior cingulate) [270], replicating the findings of a previous study [275,276]. Similar results were also found in saliva of patients with these disorders [277]. Another gene differentially methylated in two brain regions in both disorders was the dystrobrevin binding protein gene *DTNBP1*, also found in an MWAS of frontal cortex of females with both disorders [278] and in all individuals with schizophrenia [268]. The reelin (*RLN*) gene was differentially methylated in individuals with schizophrenia using an MWAS [271], as it was for schizophrenia and bipolar disorder in a MWAS of brain regions [270], replicating previous findings [279,280]. Other potential DNA methylation biomarkers for psychoses include the human leukocyte antigen (HLA) gene *HCG9* and the serotonin transported gene *SCL6A4* (*5HTT*). *HCG9* was identified in patients with schizophrenia or bipolar disorder in an MWAS of frontal cortex [278] and in brain, blood and sperm in an MWAS for bipolar disorder [281]. *SLC6A4* was differentially methylated in an MWAS of saliva and frontal cortex in individuals with schizophrenia [272], similar to previous findings in lymphoblastoid cell lines and brain tissue of individuals with bipolar disorder in a study that included cross-cohort replication [282]. No studies have investigated the possibility of using above associative biomarkers as potential risk biomarkers in early life. However, a subset of studies has found associations between DNA methylation and medication for schizophrenia or bipolar disorder [273,276]. Clearly, there is much promise for future potential biomarkers of risk, diagnosis and prognosis in schizophrenia and bipolar disorder.

More longitudinal studies at stages of life are required to generate DNA methylation biomarkers for exposure and outcome in chronic diseases other than cancer. Birth cohorts and the retrospective utility of birth dried blood spot Guthrie cards [283] will be essential for this search.

### 8.5. DNA Methylation Biomarkers of Aging

A number of individual MWAS have looked at the relationship of DNA methylation and aging, with the intention of developing age-specific biomarkers for forensic applications and for investigating premature cellular aging. Three independent meta-analyses have been performed on such datasets [284,285,286]. The first [284] reviewed six MWAS datasets from Infinium HM27 arrays containing probes for about 27,000 CpG sites [287] on a variety of cell types. None of the 1,093 age-associated probe CpG dinucleotides replicated across all six studies. However, probes at two genes, neuronal pentraxin II (*NPTX2*) and phosphodiesterase 4C (*PDE4C*), did overlap in five of the six studies. The second study [285] performed an analysis of DNA methylation from whole blood from 575 individuals ranging from newborns to age 78 from published HM27 datasets and replicated with a further group of four similar datasets. This yielded 99 significantly age-associated probes including the same *PDE4C* CpG probe cg17861230 as the first study. An even more extensive study of 39 “training” and 32 “test” HM27 and HM450 datasets of more than 7,000 samples from multiple tissues yielded 353 “age predictor” CpG dinucleotides, which included one (cg13899108) in *PDE4C* [286] just 420 bp from the CpG site identified in the first two studies. Although this locus is the most validated age-related CpG dinucleotide, these analyses show that sometimes, a combination of several CpG dinucleotides may be more accurate than a single CpG site. A recent large single analysis measured age-associated DNA methylation in whole blood DNA from 656 individuals using HM450 arrays [288]. In this tissue, investigators identified 70,387 significant age-associated CpG dinucleotides, of which, 53,670 were replicated in an independent dataset. Data was not available to identify whether the *PDE4C* locus mentioned above was among this dataset. The study went on to develop a predictive model of aging that included methylation data and clinical parameters such as gender and body mass index. We predict that this is how most DNA methylation biomarkers will be used in the future. The model selected a set of 71 age-associated methylation biomarkers that were highly predictive of age. Although *PDE4C* was not among this subset, another probe within the subset, cg09809672 associated with the EDAR-associated death domain (*EDARADD*) gene was also identified as age-associated in two of the other studies [285,286]. Importantly, this study also found evidence of an accelerated epigenetic aging in tumor tissue [288] and a further study has since identified epigenetic age acceleration as a risk factor for mortality [289]. Clearly, age-associated DNA methylation biomarkers have more applications than forensic medicine.

## 9. Integrating Epigenetic Data into Disease Risk Models

Although DNA methylation biomarkers can be used by themselves, the emerging field of molecular pathological epidemiology proposes that they can be integrated into models of disease risk together with other factors [4,290,291]. Such factors include transcriptomic, proteomic, metabolomic, microbiome, and neuroimaging data. The logic is that combinations of risk biomarkers will provide more accurate estimation of disease risk, particularly when dealing with individuals, due to inter- and intra-individual biological variation. Based on principles similar to systems and network biology and a variety of modeling methods, this field is in its infancy but is the next logical step for DNA methylation biomarkers and is already yielding promising results for genetic biomarkers [292].

## 10. Future Prospects

An increasing tendency to harmonize appropriate methods for DNA methylation detection and reference standards will accelerate the development of DNA methylation biomarkers for cancer and for other diseases. This tendency will be synergistically enhanced by next generation sequencing methodology, which has unlocked a new area of possibilities. This relatively new methodology opens the avenue for routine testing of DNA methylation biomarker panels rather than the selective choice of individual biomarkers. The use of appropriate DNA methylation biomarker panels will prove beneficial where the disease phenotype is quite heterogeneous. It is also expected that the genetic component of disease will be further revealed, which will subsequently allows the strengthening of biomarker panels by combining genetic and DNA methylation biomarker panels [293].

It is not only important to have appropriate epi(genetic) biomarker panels available for certain diseases or risk stratification but also to translate them into clinical actionable information. If no clinical action is available there is a risk of adverse psychological impacts among patients and a risk of those patients being disadvantaged by healthcare providers. However, there is also an enormous potential that affected patients can use the knowledge to their benefit allowing them to actively prevent or delay the early onset of certain diseases.

## 11. Conclusions

DNA methylation biomarkers are promising and valuable biomarkers which are heading for the molecular diagnostic laboratory. This is particular true for methylation biomarkers in cancer where the biomarkers are currently being used for early detection. However, the uptake of DNA methylation biomarkers is quite slow and will still require a considerable amount of time until the field reaches its full potential. The development of DNA methylation biomarkers for cancer and other diseases has also been slowed down by the lack of standardized methodologies and reference standards for use in DNA methylation detection. The still widespread use of inappropriate methods in combination with inappropriate controls still produces potential DNA methylation biomarkers, which may not be replicated. The need for methods of quantitative DNA methylation detection is becoming more and more obvious and is critical where only small differences in methylation values determine a diseased or disease-free state. Finally, the availability of DNA methylation biomarkers in diseases other than cancer is still in its very early steps but in time, their transition to a clinical setting will follow as it has for cancer.
